# Accuracy of Prenatal Ultrasonography for Diagnosis of Placenta Accreta Spectrum and Risk Factors in A Tertiary Center in Southern Iran

**DOI:** 10.31661/gmj.v13i.3316

**Published:** 2024-05-14

**Authors:** Homeira Vafaei, Neda Hadipour, Maryam Kasraeian, Sedigeh Yoosefi, Shaghayegh Moradi Alamdarloo, Nasrin Asadi, Zahra Oveisi, Hossein Bahari, Marjan Zare, Khadije Bazrafshan

**Affiliations:** ^1^ Maternal-Fetal Medicine Research Center, Department of Obstetrics and Gynecology, School of Medicine, Shiraz University of Medical Sciences, Shiraz, Iran; ^2^ Maternal-Fetal Medicine Research Center, Perinatology Department, Shiraz University of Medical Sciences, Shiraz, Iran 3 Student; ^3^ Student Research Committee, Shiraz University of Medical Sciences, Shiraz, Iran; ^4^ Maternal-Fetal Medicine Research Center, Shiraz University of Medical Sciences, Shiraz, Iran

**Keywords:** Placenta Accrete Spectrum, Palenta Previa, Cesarean Section, Outcome Assessment

## Abstract

Background: Placenta accreta spectrum is one of the most important causes of massive bleeding in the peripartum period. The aim of this study was to determine the accuracy of prenatal ultrasonography for diagnosis of placenta accreta spectrum (PAS) and important risk factors of this pathology were evaluated in this report. Materials and Methods: This is a cross-sectional study conducted at Shiraz University of Medical Sciences during January 2018 to January 2019. All patients who were referred for ultrasound examination of placenta accrete spectrum and surgery in Hafez tertiary center were included. Patients with diagnosis of PAS in pathology were in one group and the others in the second group. All maternal and neonatal and demographic data and surgery complications were gathered in a data form. Results: Ultrasonography was 100% (95% C.I: 94.40%-100%) sensitive, 87.58% (95% C.I; 81.29%-92.36) specific, and 87.58% (95% C.I: 82.44%-91.66%) accurate discriminating PAS from non-PAS patients. From 217 patients, 64 and 153 patients were in PAS and non-PAS group, respectively. There was significantly more age, gravidity, live children, history of DC, hormonal contraception, and history of previa in PAS group compared with Non-PAS group (p-value0.05 for all); however, gestational age was significantly lower in PAS group (p-value0.05). The odds of PAS significantly increase with previa and low-lying placenta OR adj (95% C.I): 114.68 (28.45-462.29). The patients with one C/S OR adj (95% C.I): 29.07(3.80-222.33) and the patients with two C/S OR adj (95% C.I): 106.08(13.79-815.51) were significantly more in PAS group compared with those with no C/S (p-value 0.05 for both). Conclusion: Detection rate of ultrasound examination was good, and it is recommended for women with PAS risk factors. Decreasing the rate of cesarean section and encouraging vaginal birth after cesarean section (VBAC) are the best ways of prevention of this pathology.

## Introduction

Increasing the incidence of 3 cases of placenta accreta spectrum (PAS) per 1000 pregnant women during the previous years in American society of Maternal-Fetal Medicine is terrible, and it is also on the increase [1][2]. A life-threatening post-partum hemorrhage following an increasing growth in PAS is an important problem, especially in developing countries. Failure to deliver the placenta spontaneously is defined as PAS, which has different types based on the depth of invasion [3]. Antenatal diagnosis and surgery in multidisciplinary center are the most important points in optimal management of the maternal and neonatal outcome [4].

Surgery in multidisciplinary systems with a high use of blood products, advanced intensive care units, and prolonged hospitalization impose high costs on the health care systems in high income countries; it also influences the family and fetus. In addition to the disadvantages, it causes higher mortality and morbidity in low-income countries [5].

Systematic reviews and meta-analyses have reported that ultrasonography and magnetic resonance imaging (MRI) are used for preoperational diagnosis of PAS. Both have a high detection rate, but ultrasonography is more available, less expensive and the best choice for the patients with lower financial support. Therefore, it is a good screening method. If hysterectomy is planned, depth of invasion and topography are important, and there is a controversy on which one can be diagnostic for distinguishing the types of PAS: placentas accrete, increta, or percreta [6][7][8].

Early recognition of the condition may improve the outcome. That is because it provides the obstetrician with the opportunity to deal more effectively with this obstetrical emergency. However, most cases of placenta accrete have no preceding symptoms. Therefore, higher levels of suspicion for its early diagnosis should rely on the known risk factors. Placenta previa, scarred uterus, previous uterine surgery, previous uterine curettage, advanced maternal age, and multiparity were suggested to be associated with a higher incidence of placenta accrete [9].

The aim of this study was to evaluate the accuracy of ultrasound in diagnosis of PAS and some risk factors of this fatal pathology in Iran, a country with high rate of cesarean sections and the growth of this pathology during last years.

## Materials and Methods

This cross-sectional study was conducted from January 2018 to January 2019. The pregnant women that referred for placenta ultrasound examination to Hafez High Risk Prenatal Clinic affiliated to Shiraz University of Medical Science were included in the study after signing a written informed consent. The study was approved by the ethics committee of Shiraz University of Medical Sciences (IR.SUMS.REC.1397.737). The patients who did not agree to participate in the study received the same care.

Inclusion and Exclusion Criteria

Inclusion criteria were pregnant women suspected with PAS who had diagnostic imaging and surgery in our tertiary center and those who signed informed consent to participate in the study. Exclusion criterion were the women who had undergone surgery in another center and the did not follow in this study.

Primary and Secondary Measures

Ultrasonography examination of the placenta was performed by a perinatologist and in cases the diagnosis could not be confirmed, MRI was performed.

The participants were evaluated with systematic ultrasound in their first, second or third trimesters of pregnancy (between 12 and 38weeks), using two-dimensional (2D) gray-scale imaging and color Doppler. We used a 2D ultrasound examination with abdominal and vaginal transducer (GE Voluson E6, GE Medical Systems, Austria).

PAS was diagnosed based on standard ultrasonography findings; in some cases, MRI was recommended. These findings were multiple and irregular lacunae, loss of the hypo-echoic layer between the placental-uterine interface, thinning of the myometrium, irregularity of the bladder wall, uterovesical and sub-placental hpervascularity, bridging vessels, placental buldge, exophytic mass, and placental lacunae feeder vessels [10]. Women who referred for PAS examination after giving informed consent, were enrolled in the study. Women with PAS in pathology report were selected as one group, and the others were in the second group. The perinatologist who performed ultrasound examination, the radiologist who reported MRI, and the multidisciplinary team who were involved in surgery were blind about this study. A data collection form was designed and filled by a perinatologist who had no role in diagnosis and surgery.

Patients were admitted several days before the planned cesarean hysterectomy at 34-35 weeks of gestation to stabilize the existing comorbid medical conditions, administer corticosteroids for fetal lung maturation, perform necessary evaluations, and ensure preoperative preparation. Women with vaginal bleeding or signs of labor before the scheduled time were admitted, and timing of delivery in them was based on clinical assessment of the condition. Women with focal PAS were scheduled for termination at 36-37 weeks of gestation. The patients were followed until termination of pregnancy, either planned or urgent cesarean hysterectomy. The patient was transferred to a general hospital the day before termination. The operation was done by a multidisciplinary team consisting of a gynecologist, oncologist, perinatologist or obstetrician, anesthesiologist, vascular surgeon, urologist, and neonatologist. After general anesthesia, the abdominal wall was opened by a vertical incision and the uterus by classic incision. If the placenta had adhesion and was not delivered, hysterectomy was done. The sample of hysterectomy was sent for pathology ward to get a definite diagnosis.

Statistical Analyses

Frequency (relative frequency) and mean ± sd were used to describe qualitative and quantitative variables, respectively. Kolmogorov-Smirnov test of normality, Mann-Whitney U test, Chi-Square test, Fisher’s Exact Test; binary generalized linear model were used to analyze the data, and finally, the adjusted Odds Ratio (95% confidence Interval): ORadj (95% C.I). IBM SPSS Statistics for Windows, version 23 (IBM Corp., Armonk, N.Y., USA) software tool was used at significance level<0.05 for all tests. The accuracy of the tests were evaluated using MedCalc v 20.015. The sensitivity was defined as the probability that a test result will be positive when the disease is present (true positive rate). The specificity was defined as the probability that a test result will be negative when the disease is not present (true negative arte). The positive predictive value (PPV) was defined as the probability that the disease is present when the test is positive. The negative predictive value (NPV) was defined as the probability that the disease is not present when the test is negative. And the accuracy was defined as the overall probability that a patient is correctly classified [11][12].

## Results

**Table:** Table1. Pathology and Ultrasonography Diagnosis of Placenta Accreta Spectrum Among 217 Patients.

Examination			Pathology	
		PAS	no PAS	total
	PAS	64	19	83
Ultrasonography	no PAS	0	134	134
	Total	64	153	217

**PAS:** placenta accreta spectrum

**Table:** Table2. The Sensitivity, Specificity, Positive Predictive Value, Negative Predictive Value, and Accuracy of PAS with their (95% C.I) based on Ultrasonography Diagnosis in 217 Patients.

Statistic	value	95% C.I
Sensitivity	100%	94.4%-100%
Specificity	87.58%	81.29%-92.36
Positive predictive value	0.08%	0.05%-0.12%
Negative predictive value	100%	97.28%-100%
Accuracy	87.58%	82.44%-91.66%

95% C.I, 95% confidence interval

From 217 patients, 64 and 83 patients were PAS patients based on pathology and ultrasonography examinations, respectively (Table-1). 64 and 153 patients were included in PAS and non-PAS groups, respectively. The sensitivity, specificity, PPV, NPV, and accuracy of PAS ultrasonography diagnosis with their (95% C.I) regarding PAS prevalence of 0.01 have been presented in Table-2. Ultrasonography was 100% sensitive, 87.58% specific, and 87.58% accurate discriminating PAS from non-PAS patients.

Demographic features of 64 PAS and 153 no PAS patients have been compared in Table-3.

There were significantly more age, gravidity, live children, history of D&C, hormonal contraception (Oral contraception and DMPA), and history of previa in PAS group compared with Non-PAS group (P-value<0.05 for all); however, gestational age was significantly lower in PAS group (P-value<0.05). In addition, BMI, abortion, stillbirth, interval between the last C/S, pregnancy type, and history of HTN/PEC were the same between groups (P-value>0.05 for all). The ultrasound criteria in antenatal diagnosis of 64-placenta accreta spectrum and 153 non- placenta accreta spectrum patients have been compared in Table-4.

Loss of clear zone, myometrial thinning, abnormal placenta lacuna, bladder wall interruption, bulge placenta, uterovesical hypervascularity, subplacental hypervascularity, bridging vessels, and palcental_lacunae feeder vessels were significantly higher in PAS group compared with non-PAS group (P-value<0.05 for all); however, focal exophytic mass and parametrial involvement did not differ (P-value >0.05 for both). The maternal serum markers among 64-placenta accreta spectrum and 153 non- placenta accreta spectrum patients have been compared in Table-5.

PAPPA and MSAFP were significantly higher in PAS group (P-value<0.05 for both); however, free βhCG, UE3, HCG, and Inhibin-A did not differ (P-value>0.05 for all).

The complications among 64 PAS and 153 non- PAS patients have been compared in Table-6.

Time of surgery, packed cell, ICU admission, and surgery complication were significantly higher in PAS group compared with non-PAS group (P-value<0.05 for all).

The association between the placenta type and the number of previous cesarean section among 64 PAS and 153 non- PAS patients have been shown in Table-7.The odds of PAS significantly increase with previa and low-lying placenta and the number if C/S (P-value<0.05 for both); the odds of PAS would increase by 106.08 in patients with more than two number of C/S compared with those with no C/S (P-value<0.001).

## Discussion

**Table:** Table3. Comparison Demographic Feature between 64-placenta Accreta Spectrum and 153 No Placenta Accreta Spectrum Patients

Variable	PAS group (n=64)	Non-PAS group (n=153)	P-value
Age (year), mean±sd	34.28±4.76	30.34±6.03	< 0.001 ^*^
BMI (kg/m2), mean±sd	26.77±4.75	26.23±4.86	0.46 ^*^
Gravidity, n (%)			
≤1	2(3.1%)	34(22.2%)	
2-5	57(89.1%)	117(76.5%)	<0.001 ^#^
≥5	5(7.8%)	2(1.3%)	
Live children, n (%)			
≤1	25(41.7%)	71(62.3%)	0.009 ^#^
≥2	35(58.3%)	43(37.7%)	
Abortion, n (%)			
≤1	15(55.6%)	42(66.7%)	0.32 ^#^
≥2	12(44.4%)	21(33.3%)	
Stillbirth, n (%)			
≤1	8(72.7%)	6(66.7%)	0.77 ^#^
≥2	3(27.3%)	3(33.3%)	
Interval between the last C/S (year) , mean±sd	4.97±3.16	4.32±3.02	0.15 ^*^
Gestational age at delivery time (week), mean±sd	33.48±5.38	36.43±4.16	<0.001 ^*^
History of D&C, n (%)			
Yes	12(19.4%)	12(8.1%)	0.02 ^*^
No	50(80.6%)	137(91.9%)	
Contraception, n (%)			
Hormonal	19 (29.7%)	20 (13.2%)	
Non hormonal	43 (67.2%)	109 (72.2%)	0.002 ^†^
No contraception	2 (3.1%)	22 (14.6%)	
Pregnancy type, n (%)			
ART	3(4.7%)	9(6%)	>0.99 ^†^
Spontaneous	61(95.3%)	142(94%)	
History of infertility, n (%)			
Yes	3(4.7%)	8(5.3%)	>0.99 ^†^
No	61(95.3%)	144(94.7%)	
History of HTN/PEC, n (%)			
Yes	2(3.1%)	7(4.6%)	>0.99 ^†^
No	62(96.9%)	145 (95.4%)	
History of previa, n (%)			
Yes	4(6.3%)	1(0.7%)	0.03 ^†^
No	60(93.8%)	151(99.3%)	

**PAS:** placenta accreta spectrum; BMI: body mass index; **C/S:** cesarean section; PEC: preeclampsia; D&C: dilatation and curettage; ART: Assisted reproductive technology; HTN: hypertension; *Mann-Whitney U test; # Chi-Square test; † Fisher’s Exact Test.

**Table:** Table4. Comparison of Ultrasound Criteria in Antenatal Diagnosis of 64-placenta Accreta Spectrum and 153 Non- placenta Accreta Spectrum Patients

Variable	PAS group (n=64)	Non- PAS group (n=153)	P-value
Loss of Clear zone, n (%)			
Yes	59 (95%)	3 (5%)	<0.001 ^†^
No	15 (10%)	138 (90%)	
Myometrial thinning, n (%)			
Yes	61(98.4%)	16 (10.4%)	<0.001 ^†^
No	1 (1.6%)	137(89.6%)	
Abnormal placenta lacuna, n (%)			
Yes	34 (54.8%)	6 (3.9%)	<0.001 ^#^
No	28 (45.2)	147 (96.1%)	
Bladder wall interruption, n (%)			
Yes	33 (53%)	29 (47%)	<0.001 ^#^
No	6 (3.9%)	147 (96.1%)	
Bulge placenta, n (%)			
Yes	21 (33.8%)	41 (66.2%)	<0.001 ^†^
No	4 (2.6%)	149 (97.4%)	
Focal exophytic mass, n (%)			
Yes	3 (4.9%)	1 (0.65%)	0.06 ^†^
No	58 (95.1%)	151 (99.35%)	
Uterovesical hypervascularity, n (%)			
Yes	51 (82.2%)	10 (6.53%)	<0.001 ^#^
No	11 (17.8%)	143 (93.47%)	
Subplacental hypervascularity, n (%)			
Yes	44 (70.9%)	12 (7.8%)	<0.001 ^#^
No	18 (2.91%)	141 (92.2%)	
Bridging vessels, n (%)			
Yes	45 (72.58%)	8 (5.23%)	<0.001 ^#^
No	17 (27.42)	145 (94.77%)	
palcental_lacunae feeder vessels, n (%)			
Yes	6 (9.84%)	2 (1.31%)	0.006 ^#^
No	55 (90.16%)	151 (98.69%)	
parametrial involvement, n (%)			
Yes	4 (6.45%)	0 (0%)	0.06 ^†^
No	58 (93.55%)	151 (100%)	

# Chi-Square test; † Fisher’s Exact Test; PAS: placenta accrete specterum

**Table:** Table5. Comparison of Maternal Serum Markers among 64-placenta Accreta Spectrum and 153 Non- placenta Accreta Spectrum Patients.

Screening serum markers(mean±sd)	PAS group (n=64)	No PAS group (n=153)	P-value
PAPPA,(MOM)	3.7±11.68	1.14±0.89	0.03*
Free βhCG,(MOM)	1.24±1.08	2.69±14.04	0.6*
MSAFP, (MOM)	2.06±1.87	1.24±0.51	0.03*
UE_3_, (MOM)	0.99±0.31	1.29±0.76	0.14*
HCG, (MOM)	1.15±0.96	1.2±0.83	0.91*
Inhibin-A, (MOM)	1.39±1.17	1.42±1.02	0.92[WU1] *

^*^Mann-Whitney U test; **PAS:** placenta accreta spectrum; **PAPPA:** pregnancy-associated plasma protein A; **Free βhCG:** Free Human chorionic gonadotropin; **MSAFP:** Maternal serum Alpha-fetoprotein; **UCE:** Unconjugated Estriol; **HCG:** Human chorionic gonadotropin,. **MOM:** Multiples of the normal median.

**Table:** Table6. Comparison of Complications among 64-placenta Accreta Spectrum and 153 Non- placenta Accreta Spectrum Patients

Variable	PAS group (n=64)	No PAS group (n=153)	P-value
Time of surgery (minute), mean±sd	193.83±59.48	70.98±28.78	<0.001 ^*^
Packed cell (number)(57/2), mean±sd	2.65±2.73	0.09±0.47	<0.001 ^*^
Blood loss(CC), mean±sd[WU1]	2750±2574.4	506.58±422.76	<0.001 ^*^
ICU admission, n (%)			
Yes	34 (53.12%)	2 (1.31%)	<0.001 ^†^
No	30 (46.88%)	151 (98.69%)	
Surgery complication, n (%)	20 (31.25%)	1 (0.65%)	
Ureter ligation	3 (15%)	0 (0%)	
Bladder rupture	9 (45%)	0 0%)	<0.001 ^†^
Pelvic hematoma	1 (5%)	1 (100%)	<0.001 ^†^
Hypogastric artery ligation	2 (10%)	0 (0%)	
Reoperation	5 (25%)	0(0%)	

^*^Mann-Whitney U test; † Fisher’s Exact Test; PAS: placenta accrete spectrum

**Table:** Table7. The Association between the Placenta Type and the Number of Previous Cesarean Section among 64-placenta Accreta Spectrum and 153 Non- placenta Accreta Spectrum Patients

Variable	PAS group (n=64)	Non-PAS group (n=153)	P-value	OR ^*^_adj_ (95% C.I)
Placenta, n (%)			
Previa and low-lying	59 (78.7%)	16(21.3%)	<0.001	114.68 (28.45-462.29)
Other (reference category)	5 (3.6%)	134(96.4%)		1(.-.)
Number of previous C/S			-	
0 (reference category)	1(1.6%)	67(46.9%)	<0.001	1(.-.)
1	23 (37.1%)	53(37.1%)	<0.001	29.07(3.8-222.33)
≥2	38(61.3%)	23(16.1%)		106.08(13.79-815.51)

^*^adjusted on age, gravidity, live children, gestational age at delivery, history of D&C, contraception, and history of previa; OR (95% C.I): Odds Ratio (95 % confidence interval)

Placenta accreta spectrum is one of the most important causes of massive bleeding in the peripartum period. This cross-sectional study was conducted in a tertiary center in Southern Iran. In this study, approach to PAS was multidisciplinary as reported in other studies [13]. This study reported a significantly higher rate of PAS when increasing the number of previous cesarean section, placenta previa, dilatation and curettage, age, gravidity, alive children, and history of dead fetus in the PAS group than those in the other group. Increased live birth, gravidity, and dead fetus in these patients resulted in increased number of repeated cesarean section. Thus, judging whether these variables are the main risk factors or caesarian section is indefinite. Only 2 patients with pathology of PAS had undergone no previous cesarean section. Therefore, in this sample size, we cannot highlight this result. The outcome was like another Iranian experiences, showing that PAS pregnancies managed in a resource-limited setting in Southern Iran have both maternal and neonatal outcomes comparable to those in developed countries, which is hypothesized to be due to the high rate of antenatal diagnosis (86.3%) and multidisciplinary approach used for the management of pregnancies with PAS [14]. The mean interval age between pregnancies had no significant difference between the two groups.

Although this sample size was terrible for this pathology in one year for one referral center in the south and southwest of Iran, some risk factors and criteria did not have acceptable frequency for analysis: 4 previous cesarean sections only with 2 patients, history of surgery on the uterus, uterine anomaly, previous uterine rupture, and maternal blood group. Moreover, maternal BMI, history of abortion, pregnancy with ART, history of HTN, preeclampsia, infertility, maternal serum markers in the first and second trimesters for aneuploidy screening had no significant relationship with this pathology.

Thurn et al. in 2015 reported 3 years of experience in five countries: Denmark, Finland, Iceland, Norway, and Sweden. 205 cases were diagnosed during the study; 49% of all PAS cases occurred in women with placenta previa. Seven times increased risk of PAS with one cesarean section and 56 times with two or three incisions were the other finding of this study [9]. The important result of the present study was that odds ratio in one previous cesarean section to patients without this history was 29, and patients with more than 2 previous history had 106 times higher chance for this pathology; this is a very terrible event in the obstetric field. Placenta previa increased this chance about 95 times in the present study. This result is significantly higher than that of these countries with a difference that they reported about 50% of patients with negative history of cesarean section, but in this study only 1.28% of the PAS group did not have repeated cesarean section. These two risk factors are the most important ones in Iranian population.

Marcellin et al.’s study in 2018 retrospectively reported 156 cases of PAS in 5 years, comparing the depth of invasion of placenta percreta with the others. In 51 women with percreta, significantly higher BMI, gravidity and parity, and number of previous cesarean section were reported in comparison with other types of PAS [15].

A binational case control study in Australia and New Zealand reported a significant relationship between BMI and PAS [16]; although increasing BMI with a higher rate of cesarean section can increase the chance of PAS, here this relationship was not significant.

Some studies have reported the probability of a relationship between the first trimester aneuploidy screening and second trimester MSAFP with PAS; they did not have significant relationships. The result of the present study was the same and did not confirm this theory [17][18][19]. Some studies concluded that antenatal diagnosis and surgery in elective condition could decrease the rate of complication [20][21], but in our study with antenatal diagnosis of all cases, there was no significant difference between the patients who had undergone elective surgery in 34-35 weeks of gestation or emergent cesarean due to maternal or fetal problems. One of the reasons was that all operations were performed in the hospitals affiliated to Shiraz University of Medical Sciences with surgeons and other multidisciplinary team who had adequate experience. Shamshirsaz et al. reported that PAS patients who delivered in multidisciplinary center in elective setting had lower complications[22][23].

A systematic review in 2017 reported that ultrasound examination had a good accuracy in finding PAS with a sensitivity of 81.2% and specificity of 98.9% [7]. The present report showed that in our center ultrasound diagnosis was 100% sensitive for PAS but had 21% overdiagnosis in highly suspicious patients. Due to high costs imposed on the health care system, it is important to improve the accuracy of diagnosis even though saving the mothers is the best choice.

## Conclusion

Women with risk factors of PAS should have ultrasonography examination before delivery although the surgeon should be cautious about abnormal placenta invasion. Since the cesarean section is the most effective risk factor in PAS, decreasing the rate of cesarean section is the best prevention. Also, because with increasing the number of cesarean sections this risk increases progressively, trial of labor after cesarean section is a good suggestion for patients who plan to have more than two children in their family.

## Acknowledgement

The authors would like to thank Shiraz University of Medical Sciences, Shiraz, Iran and also Center for Development of Clinical Research of Nemazee Hospital and Dr. Nasrin Shokrpour for editorial assistance. The study was funded by Shiraz University of Medical Sciences (Grant 1396-01-85-15947).

## Conflict of Interest

There is no conflict of interest to be declared regarding the manuscript.
